# Defining the Damaged DNA Mobility Paradox as Revealed by the Study of Telomeres, DSBs, Microtubules and Motors

**DOI:** 10.3389/fgene.2018.00095

**Published:** 2018-03-20

**Authors:** Karim Mekhail

**Affiliations:** ^1^Department of Laboratory Medicine and Pathobiology, University of Toronto, MaRS Centre, Toronto, ON, Canada; ^2^Canada Research Chairs Program, University of Toronto, Toronto, ON, Canada

**Keywords:** nuclear organization, DSB mobility, telomeres, DSB repair, kinesin, microtubules, chromatin remodeling, heterochromatin

## Abstract

Eukaryotic genomes are non-randomly arranged inside the nucleus. Despite this ordered spatial genome organization, damaged DNA exhibits increased random mobility within nuclear space. This increased random movement is thought to promote DNA repair by facilitating homology search, allowing targeting to repair-conducive nuclear domains, or releasing damage from repair-repressive locations. Recent studies focusing on the relationship between telomeres, DNA repair processes, and nuclear organization have revealed that the disruption of motor proteins or microtubules, which typically mediate the directed motion of cargo, disrupts the random mobility of damaged DNA. These findings define a new biological paradox. Here, I define this as the damaged DNA mobility paradox, describe how it uncovers key gaps in knowledge, and highlight key questions to help guide us toward paradox resolution.

## Background

Looking back at the history of biological research, we typically go through key stages before achieving an advanced understanding of biological processes. First, new data suggest that a process must exist. Second, the search for the process provides early clues that can even seem paradoxical. Third, new information resolves the paradox, clarifies the biological process, and helps us reach consensus. In fact, we can proceed through these three steps one or more times for a given process and uncover new knowledge with each cycle. In the steps outlined above, one cannot underestimate the importance of biological paradoxes. Such paradoxes or problems help us challenge scientific dogma, synthesize new hypotheses, develop new technologies and even create new fields of research.

One historical example of a biological paradox is known as the end replication problem (Watson, 1972; Olovnikov, 1973). Following the discovery of the double helical DNA structure and clarification of the mechanism of DNA replication, a paradox emerged (Watson, 1972; Olovnikov, 1973). It consisted of the fact that the DNA replication machinery cannot replicate the ends of linear chromosomes and yet, chromosome length was relatively stable in living cells (Watson, 1972; Olovnikov, 1973). This paradox was eventually resolved by the identification of the enzyme telomerase, which extends telomeric DNA sequences at the ends of linear chromosomes (Szostak and Blackburn, 1982; Greider and Blackburn, 1985). Since telomerase can compensate for the inability of the DNA replication machinery to replicate the very ends of linear chromosomes, paradox resolution was achieved and consensus mechanisms emerged (Wellinger, 2014). Thus, the paradox of the DNA end replication problem was resolved by the identification of telomeres and telomerase, which in turn served as the foundation of an entirely new field of research focusing on the role of telomeres in basic biology as well as human health and disease (Armanios and Blackburn, 2012; Wellinger, 2014).

Here, I will illustrate how recent studies, including research that ironically focused on telomeres, reveal a new biological paradox (Chung et al., 2015; Lottersberger et al., 2015). The paradox is pertinent to our understanding of the processes that increase the mobility of damaged DNA in order to promote its repair. Key features of this paradox will be defined before highlighting major gaps in knowledge or pending questions that need to be addressed before we can achieve paradox resolution.

## The damaged DNA mobility paradox

During the interphase stage of the cell cycle, eukaryotic genomes are non-randomly arranged inside the nucleus, which is defined by the nuclear envelope (Mekhail and Moazed, 2010; Poon and Mekhail, 2011). Moreover, this non-random organization responds to endogenous or external cues. This response is thought to be critical to genome expression and stability (Mekhail and Moazed, 2010). One striking example consists of the increased mobility of DNA in response to endogenously or exogenously induced DNA damage (Marnef and Legube, 2017). The increased mobility of damaged DNA has been observed in live yeast, worm, fly, mouse, and human cells (Lisby et al., 2003; Aten et al., 2004; Kruhlak et al., 2006; Torres-Rosell et al., 2007; Dimitrova et al., 2008; Mekhail et al., 2008; Nagai et al., 2008; Jakob et al., 2009; Khadaroo et al., 2009; Oza et al., 2009; Chiolo et al., 2011; Dion et al., 2012; Mine-Hattab and Rothstein, 2012; Saad et al., 2014; Chung et al., 2015; Lottersberger et al., 2015; Ryu et al., 2015; Strecker et al., 2016; Tsouroula et al., 2016; Aymard et al., 2017; Lawrimore et al., 2017). In these studies, the fluorescent labeling of damaged DNA or its associated DNA repair factors revealed that DNA loci subjected to a damaging event explore a larger proportion of the nucleus. This is true for telomere-proximal and telomere-distal DNA loci subjected to a double strand break (DSB) (Therizols et al., 2006; Nagai et al., 2008; Chung et al., 2015; Lottersberger et al., 2015; Strecker et al., 2016; Lawrimore et al., 2017). This is also the case for the DSB-like eroded telomeres of telomerase-deficient cells or uncapped telomeres in cells lacking telomere-capping factors (Benanti et al., 2009; Khadaroo et al., 2009; Lottersberger et al., 2015). The increased movement of damaged DNA inside the nucleus is thought to promote DNA repair by acting on one or more levels (Lisby et al., 2003; Aten et al., 2004; Kruhlak et al., 2006; Torres-Rosell et al., 2007; Dimitrova et al., 2008; Mekhail et al., 2008; Nagai et al., 2008; Jakob et al., 2009; Khadaroo et al., 2009; Oza et al., 2009; Chiolo et al., 2011; Dion et al., 2012; Mine-Hattab and Rothstein, 2012; Saad et al., 2014; Chung et al., 2015; Lottersberger et al., 2015; Ryu et al., 2015; Strecker et al., 2016; Tsouroula et al., 2016; Aymard et al., 2017; Lawrimore et al., 2017). First, increased mobility can facilitate contacts between donor and acceptor DNA sequences subject to homology-directed repair. Second, the elevated mobility can allow damaged DNA to escape repair-repressive nuclear neighborhoods such as the nucleolus or heterochromatic subnuclear domains. Third, increased motion can facilitate the relocation of damaged DNA to repair-conducive nuclear neighborhoods, which include the subnuclear domain of nuclear pore complexes (NPCs).

Importantly, while the genome is non-randomly arranged in the nucleus in the presence or absence of DNA damage, the above studies revealed that damaged DNA exhibiting an increased mobility appears to move randomly inside the nucleus (Seeber and Gasser, 2016). This randomness has been observed whether damaged DNA is directly tracked using *lacO-*LacR/*tetO-*TetR operator strategies or indirectly tracked using fluorescently labeled DNA repair proteins. This is consistent with a model in which the increased mobility of damaged DNA may be achieved via the release of physical constraints that are normally exerted onto chromatin. Consistent with this rationale, damaged DNA undergoes extensive chromatin remodeling that allows easier access of the damaged genetic information by the DNA repair machinery (Seeber and Gasser, 2016). In addition, DNA damage can promote DNA mobility by relieving physical restraints that typically act on the genome as a whole in the absence of DNA damage. For example, in the budding yeast *Saccharomyces cerevisiae*, induction of a single DSB increases the mobility of the DSB site and possibly other portions of the genome by relieving the constraints typically exerted onto chromosomes via the attachment of centromeres to the spindle pole body and the tethering of telomeres to the nuclear envelope (Strecker et al., 2016). Similar to these yeast findings, the increased mobility of damaged DNA loci, and also non-damaged DNA loci to a lesser degree, has been reported in mouse cells (Lottersberger et al., 2015). Thus, the relief of chromatin constraint and physical tethering constraints onto chromosomes is thought to passively increase damaged DNA mobility.

**Figure 1:**
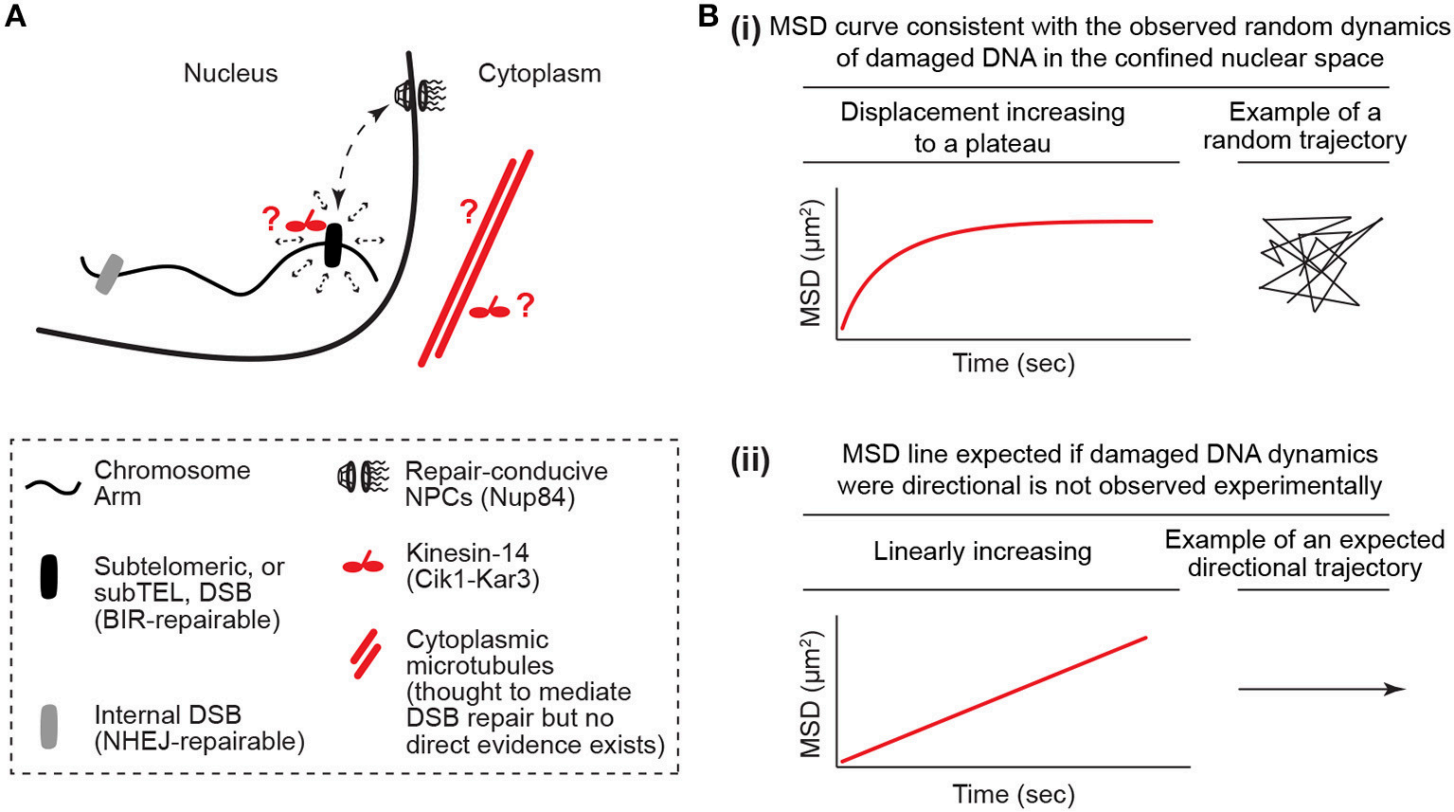
The damaged DNA mobility paradox. **(A)** Kinesin/microtubule-dependent increased random mobility and targeting of damaged DNA to repair-conducive nuclear pore complexes. The damaged DNA mobility paradox is defined by the fact that microtubules and motors, which typically mediate the directed motion of cargo, increase the random mobility of damaged DNA in the nucleus. It is also unclear how the cytoplasmic microtubules cooperate with motors in the nucleus or cytoplasm to perform this function. **(B)** Illustration of how mean square displacement (MSD) analyses reveal DNA mobility profiles that are more consistent with random mobility in a confined nuclear space and not linear directed mobility. The MSD y-axis is log scaled.

While this is logical, it cannot explain all of the literature. In particular, the results of a couple of studies focusing primarily on telomeres and telomere-proximal regions known as subtelomeres challenge models in which the loss of genomic constraint passively promotes the mobility of damaged DNA (Chung et al., 2015; Lottersberger et al., 2015). In *S. cerevisiae*, repair of a single inducible subtelomeric DSB is dependent on the NPC subcomplex NUP84, which is composed of seven subunits including the Nup84 protein (Therizols et al., 2006; Chung et al., 2015; Oshidari and Mekhail, 2018). In addition, induction of these subtelomeric DSBs results in an increased physical interaction of the DSB site with Nup84, which is thought to promote repair through its association with DNA repair-conducive factors such as the Slx5-Slx8 protein complex (Nagai et al., 2008; Chung et al., 2015). Intriguingly, deletion of the microtubule-stabilizing α-tubulin isoform Tub3 also compromised subtelomeric DSB repair (Chung et al., 2015). In addition, DSB repair was found to be dependent on a motor protein complex called Kinesin-14, which is composed of the catalytic subunit Kar3 and structural subunit Cik1 (Chung et al., 2015). Moreover, Kinesin-14 disruption decreased the physical association of the subtelomeric DSB with Nup84 in chromatin immunoprecipitation experiments (Figure 1A; Chung et al., 2015). Microtubules and kinesin motors typically mediate the directed motion of cargo. In contrast, analysis of the mobility of subtelomeric DSBs using a type of single particle motion analysis called mean square displacement (MSD) paradoxically revealed that these subtelomeric DSBs, similar to DSBs induced at other locations across the genome, move randomly inside the nucleus (Figure 1B; Chung et al., 2015). Therefore, although these subtelomeric DSBs rely on microtubules and motors for repair, these DSB sites exhibit random mobility within nuclear space. Moreover, similar findings were reported in mouse cells. Specifically, upon disruption of telomere capping, telomeres behave like DSB ends and exhibit an increased mobility inside the nucleus (Dimitrova et al., 2008; Lottersberger et al., 2015). This mobility drives telomere fusion repair of uncapped telomeres via the non-homologous end-joining pathway. Paradoxically, the increased mobility and fusion repair of uncapped telomeres is compromised via knockdown of Kinesin-1 or Kinesin-2, or upon treatment with the microtubule stabilizer taxol or microtubule depolymerizing nocodazole (Lottersberger et al., 2015). In addition, no directed motion was observed for these DSB-like uncapped telomeres. Moreover, both in yeast and murine cells, damaged DNA outside of telomeres also appeared to exhibit increased random mobility and repair that is dependent on microtubules or kinesin motor proteins (Chung et al., 2015; Lottersberger et al., 2015). The key unifying feature appeared to be the type of DNA repair pathway that is used. For example, in yeast, telomeric and non-telomeric DNA breaks that are repairable by the homologous recombination pathway subtype break-induced replication (BIR) exhibited dependence on microtubules and motors for DNA repair. This suggests that lessons learned from the study of damaged or uncapped telomeric regions may be widely applicable across genomes. Taken together, the reported roles for microtubules and molecular motors, which typically mediate the directed transport of cargo, in DSB mobility and repair are paradoxical when considered in light of the observation that damaged DNA moves randomly inside nuclei.

## Toward paradox resolution

**Figure 2:**
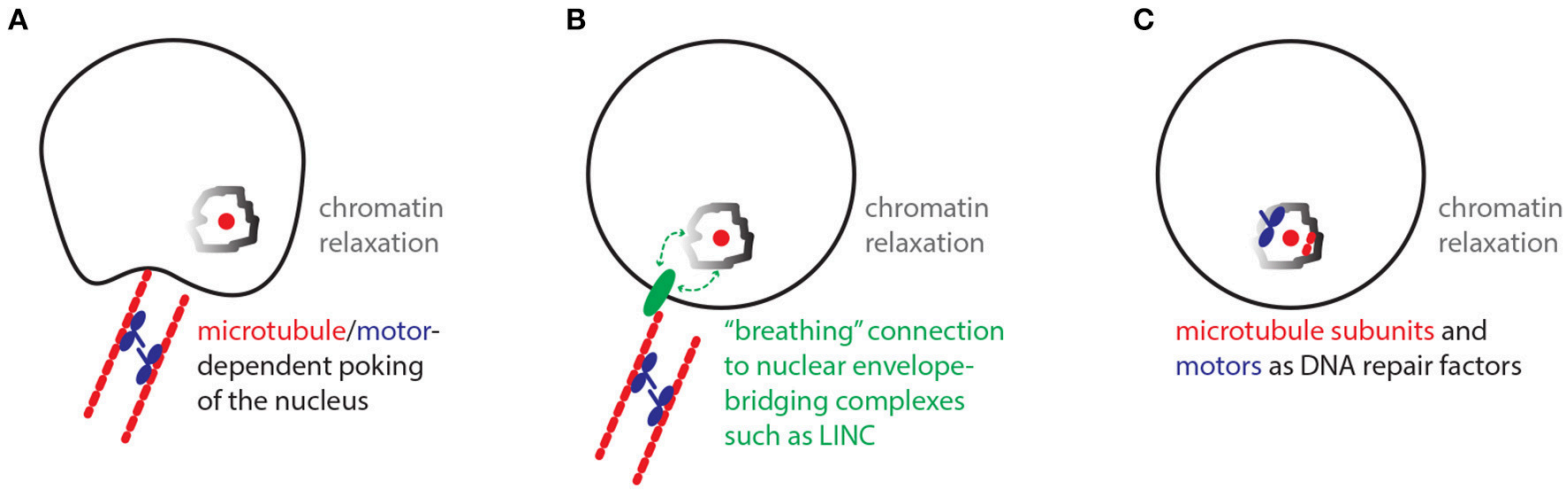
Models with the potential to resolve the damaged DNA mobility paradox. **(A)** Nuclear poking model. In the cytoplasm, microtubules/motors-dependent poking of the nuclear envelope promotes the mobility of chromatin especially around damaged DNA sites, where chromatin remodeling/relaxation is known to occur. **(B)** Breathing LINC model. The relaxed chromatin around damaged DNA sites may be loosely interacting with Linker of Nucleoskeleton and Cytoskeleton (LINC) protein complexes, which can be mobilized along the nuclear envelope via connections to cytoplasmic microtubules and motors. **(C)** Repair factors model. Microtubule subunits and motors may exert non-traditional roles and promote damaged DNA mobility and repair by directly promoting the function of chromatin remodeling complexes or other repair-promoting factors.

Similar to the historical paradox of the end replication problem, the damaged DNA mobility paradox defined here challenges our current models of damaged DNA mobility and repair (Watson, 1972; Chung et al., 2015; Lottersberger et al., 2015). While the paradox of the end replication problem has long been resolved via the discovery of telomeres and telomerase, the damaged DNA mobility paradox remains a mystery. So which models could solve this paradox? In one model, motors may regulate microtubules in the cytoplasm in order to physically “poke” the nucleus and randomly mobilize nuclear chromatin (Figure 2A; Lottersberger et al., 2015). Despite the potential of such processes to increase overall genome dynamics, the random mobility of DSB sites may be especially increased by global nuclear poking when it is combined with the known relaxation of chromatin restraint at damaged DNA sites. In another model, cytoplasmic microtubules and motors may help mobilize damaged chromatin inside the nucleus via interactions with nuclear envelope-bridging protein complexes such as the Linker of Nucleoskeleton and Cytoskeleton (LINC) complex (Lottersberger et al., 2015) (Figure 2B) (Tapley and Starr, 2013). In this case, the association of chromatin with nuclear envelope-bridging complexes may be transient in nature allowing for a “breathing” random motion of chromatin. Consistent with this model, the mobility and fusion repair of uncapped telomeres in mammalian cells is dependent on the LINC complex (Lottersberger et al., 2015). An additional model stipulates that motor proteins and microtubule subunits may operate more like DNA repair proteins that may for example help relax chromatin structure in the nucleus (Figure 2C). In this scenario, motor and microtubule proteins would not be exerting a traditional transport of cargo role. Consistent with this possibility, pull-down experiments revealed that yeast Kinesin-14 is physically recruited to subtelomeric DSB sites via interaction with the perinuclear tethering complex cohibin (Chan et al., 2011; Poon and Mekhail, 2012; Chung et al., 2015). In addition, in human cells subjected to ionizing radiation, the kinesin KIF4A is co-enriched with BRCA2 at DSB sites and KIF4A loss compromises the formation of Rad51 DNA repair foci (Wu et al., 2008). Moreover, ionizing radiation-induced damaged DNA foci exhibit increased mobility that is dependent on kinesins and microtubules (Lottersberger et al., 2015). In addition to the above-described models, we can also rule out some models. For example, the possibility that the nuclear motors may be cooperating with nuclear actin to mediate yeast subtelomeric DSB repair and murine telomere fusion repair is unlikely since the disruption of actin filaments does not compromise the damaged DNA mobility or repair in these contexts (Chung et al., 2015; Lottersberger et al., 2015; Lawrimore et al., 2017). In addition, while actin-related processes can affect DNA repair, it is unclear if these processes involve the increased mobility of DNA or even implicate molecular motors (Belin et al., 2015).

A number of key questions need to be addressed in order to improve our understanding of damaged DNA mobility-dependent repair and help resolve the damaged DNA mobility paradox. Do all DNA breaks exhibit increased random mobility? Is there a possibility that unknown confounding factors cause the mobility of damaged DNA to appear random when it is not? In different cell populations, tissues or species, what are the similarities and differences in the types of motion exhibited by damaged DNA and the nature of the processes mediating DNA mobility-dependent repair?

All in all, by defining the damaged DNA mobility paradox, understanding how it uncovers key gaps in knowledge, and pointing to possible avenues through which the paradox may be resolved, I hope that this perspective will help us resolve yet another mysterious, yet compelling, paradox.

## Author contributions

The author confirms being the sole contributor of this work and approved it for publication.

### Conflict of interest statement

The author declares that the research was conducted in the absence of any commercial or financial relationships that could be construed as a potential conflict of interest.

## References

[B1] Armanios M. Blackburn E. H. (2012). The telomere syndromes. Nat. Rev. Genet. 13, 693–704. 10.1038/nrg3246 22965356 PMC3548426

[B2] Aten J. A. Stap J. Krawczyk P. M. Van Oven C. H. Hoebe R. A. Essers J.. (2004). Dynamics of DNA double-strand breaks revealed by clustering of damaged chromosome domains. Science 303, 92–95. 10.1126/science.1088845 14704429

[B3] Aymard F. Aguirrebengoa M. Guillou E. Javierre B. M. Bugler B. Arnould C.. (2017). Genome-wide mapping of long-range contacts unveils clustering of DNA double-strand breaks at damaged active genes. Nat. Struct. Mol. Biol. 24, 353–361. 10.1038/nsmb.3387 28263325 PMC5385132

[B4] Belin B. J. Lee T. Mullins R. D. (2015). DNA damage induces nuclear actin filament assembly by Formin−2 and Spire-(1/2) that promotes efficient DNA repair. Elife 4:e07735 10.7554/eLife.07735 26287480 PMC4577826

[B5] Benanti J. A. Matyskiela M. E. Morgan D. O. Toczyski D. P. (2009). Functionally distinct isoforms of Cik1 are differentially regulated by APC/C-mediated proteolysis. Mol Cell 33, 581–590. 10.1016/j.molcel.2009.01.032 19285942 PMC2883288

[B6] Chan J. N. Poon B. P. Salvi J. Olsen J. B. Emili A. Mekhail K. (2011). Perinuclear cohibin complexes maintain replicative life span via roles at distinct silent chromatin domains. Dev Cell 20, 867–879. 10.1016/j.devcel.2011.05.014 21664583

[B7] Chiolo I. Minoda A. Colmenares S. U. Polyzos A. Costes S. V. Karpen G. H. (2011). Double-strand breaks in heterochromatin move outside of a dynamic HP1a domain to complete recombinational repair. Cell 144, 732–744. 10.1016/j.cell.2011.02.012 21353298 PMC3417143

[B8] Chung D. K. Chan J. N. Strecker J. Zhang W. Ebrahimi-Ardebili S. Lu T.. (2015). Perinuclear tethers license telomeric DSBs for a broad kinesin- and NPC-dependent DNA repair process. Nat. Commun. 6:7742. 10.1038/ncomms8742 26205667

[B9] Dimitrova N. Chen Y. C. Spector D. L. de Lange T. (2008). 53BP1 promotes non-homologous end joining of telomeres by increasing chromatin mobility. Nature 456, 524–528. 10.1038/nature07433 18931659 PMC2613650

[B10] Dion V. Kalck V. Horigome C. Towbin B. D. Gasser S. M. (2012). Increased mobility of double-strand breaks requires Mec1, Rad9 and the homologous recombination machinery. Nat. Cell Biol. 14, 502–509. 10.1038/ncb2465 22484486

[B11] Greider C. W. Blackburn E. H. (1985). Identification of a specific telomere terminal transferase activity in Tetrahymena extracts. Cell 43, 405–413. 10.1016/0092-8674(85)90170-9 3907856

[B12] Jakob B. Splinter J. Durante M. Taucher-Scholz G. (2009). Live cell microscopy analysis of radiation-induced DNA double-strand break motion. Proc. Natl. Acad. Sci. U.S.A. 106, 3172–3177. 10.1073/pnas.0810987106 19221031 PMC2642473

[B13] Khadaroo B. Teixeira M. T. Luciano P. Eckert-Boulet N. Germann S. M. Simon M. N.. (2009). The DNA damage response at eroded telomeres and tethering to the nuclear pore complex. Nat. Cell Biol. 11, 980–987. 10.1038/ncb1910 19597487

[B14] Kruhlak M. J. Celeste A. Dellaire G. Fernandez-Capetillo O. Müller W. G. McNally J. G.. (2006). Changes in chromatin structure and mobility in living cells at sites of DNA double-strand breaks. J. Cell Biol. 172, 823–834. 10.1083/jcb.200510015 16520385 PMC2063727

[B15] Lawrimore J. Barry T. M. Barry R. M. York A. C. Friedman B. Cook D. M.. (2017). Microtubule dynamics drive enhanced chromatin motion and mobilize telomeres in response to DNA damage. Mol. Biol. Cell 28, 1701–1711. 10.1091/mbc.E16-12-0846 28450453 PMC5469612

[B16] Lisby M. Mortensen U. H. Rothstein R. (2003). Colocalization of multiple DNA double-strand breaks at a single Rad52 repair centre. Nat. Cell Biol. 5, 572–577. 10.1038/ncb997 12766777

[B17] Lottersberger F. Karssemeijer R. A. Dimitrova N. De Lange T. (2015). 53BP1 and the LINC complex promote microtubule-dependent DSB Mobility and DNA Repair. Cell 163, 880–893. 10.1016/j.cell.2015.09.057 26544937 PMC4636737

[B18] Marnef A. Legube G. (2017). Organizing DNA repair in the nucleus: DSBs hit the road. Curr Opin Cell Biol 46, 1–8. 10.1016/j.ceb.2016.12.003 28068556

[B19] Mekhail K. Moazed D. (2010). The nuclear envelope in genome organization, expression and stability. Nat. Rev. Mol. Cell Biol. 11, 317–328. 10.1038/nrm2894 20414256 PMC3246372

[B20] Mekhail K. Seebacher J. Gygi S. P. Moazed D. (2008). Role for perinuclear chromosome tethering in maintenance of genome stability. Nature 456, 667–670. 10.1038/nature07460 18997772 PMC2596277

[B21] Miné-Hattab J. Rothstein R. (2012). Increased chromosome mobility facilitates homology search during recombination. Nat. Cell Biol. 14, 510–517. 10.1038/ncb2472 22484485

[B22] Nagai S. Dubrana K. Tsai-Pflugfelder M. Davidson M. B. Roberts T. M. Brown G. W.. (2008). Functional targeting of DNA damage to a nuclear pore-associated SUMO-dependent ubiquitin ligase. Science 322, 597–602. 10.1126/science.1162790 18948542 PMC3518492

[B23] Olovnikov A. M. (1973). A theory of marginotomy. The incomplete copying of template margin in enzymic synthesis of polynucleotides and biological significance of the phenomenon. J. Theor. Biol. 41, 181–190. 10.1016/0022-5193(73)90198-7 4754905

[B24] Oshidari R. Mekhail K. (2018). Assays to study repair of inducible DNA double-strand breaks at telomeres. Methods Mol. Biol. 1672, 375–385. 10.1007/978-1-4939-7306-4_26 29043637

[B25] Oza P. Jaspersen S. L. Miele A. Dekker J. Peterson C. L. (2009). Mechanisms that regulate localization of a DNA double-strand break to the nuclear periphery. Genes Dev. 23, 912–927. 10.1101/gad.1782209 19390086 PMC2675867

[B26] Poon B. P. Mekhail K. (2011). Cohesin and related coiled-coil domain-containing complexes physically and functionally connect the dots across the genome. Cell Cycle 10, 2669–2682. 10.4161/cc.10.16.17113 21822055 PMC3219537

[B27] Poon B. P. Mekhail K. (2012). Effects of perinuclear chromosome tethers in the telomeric URA3/5FOA System reflect changes to gene silencing and not nucleotide metabolism. Front. Genet. 3:144 10.3389/fgene.2012.00144 22876257 PMC3410493

[B28] Ryu T. Spatola B. Delabaere L. Bowlin K. Hopp H. Kunitake R.. (2015). Heterochromatic breaks move to the nuclear periphery to continue recombinational repair. Nat. Cell Biol. 17, 1401–1411. 10.1038/ncb3258 26502056 PMC4628585

[B29] Saad H. Gallardo F. Dalvai M. Tanguy-Le-Gac N. Lane D. Bystricky K. (2014). DNA dynamics during early double-strand break processing revealed by non-intrusive imaging of living cells. PLoS Genet. 10, e1004187. 10.1371/journal.pgen.1004187 24625580 PMC3952824

[B30] Seeber A. Gasser S. M. (2016). Chromatin organization and dynamics in double-strand break repair. Curr. Opin. Genet. Dev. 43, 9–16. 10.1016/j.gde.2016.10.005 27810555

[B31] Strecker J. Gupta G. D. Zhang W. Bashkurov M. Landry M. C. Pelletier L.. (2016). DNA damage signalling targets the kinetochore to promote chromatin mobility. Nat. Cell Biol. 18, 281–290. 10.1038/ncb3308 26829389

[B32] Szostak J. W. Blackburn E. H. (1982). Cloning yeast telomeres on linear plasmid vectors. Cell 29, 245–255. 10.1016/0092-8674(82)90109-X 6286143

[B33] Tapley E. C. Starr D. A. (2013). Connecting the nucleus to the cytoskeleton by SUN-KASH bridges across the nuclear envelope. Curr. Opin. Cell Biol. 25, 57–62. 10.1016/j.ceb.2012.10.014 23149102 PMC3578026

[B34] Therizols P. Fairhead C. Cabal G. G. Genovesio A. Olivo-Marin J. C. Dujon B.. (2006). Telomere tethering at the nuclear periphery is essential for efficient DNA double strand break repair in subtelomeric region. J. Cell Biol. 172, 189–199. 10.1083/jcb.200505159 16418532 PMC2063549

[B35] Torres-Rosell J. Sunjevaric I. De Piccoli G. Sacher M. Eckert-Boulet N. Reid R.. (2007). The Smc5-Smc6 complex and SUMO modification of Rad52 regulates recombinational repair at the ribosomal gene locus. Nat. Cell Biol. 9, 923–931. 10.1038/ncb1619 17643116

[B36] Tsouroula K. Furst A. Rogier M. Heyer V. Maglott-Roth A. Ferrand A.. (2016). Temporal and Spatial Uncoupling of DNA Double Strand Break Repair Pathways within Mammalian Heterochromatin. Mol. Cell 63, 293–305. 10.1016/j.molcel.2016.06.002 27397684

[B37] Watson J. D. (1972). Origin of Concatemeric T7DNA. Nat. New Biol. 239:197. 10.1038/newbio239197a0 4507727

[B38] Wellinger R. J. (2014). In the end, what's the problem? Mol. Cell 53, 855–856. 10.1016/j.molcel.2014.03.008 24656125

[B39] Wu G. Zhou L. Khidr L. Guo X. E. Kim W. Lee Y. M.. (2008). A novel role of the chromokinesin Kif4A in DNA damage response. Cell Cycle 7, 2013–2020. 10.4161/cc.7.13.6130 18604178 PMC3121316

