# Corrigendum: Defining the Damaged DNA Mobility Paradox as Revealed by the Study of Telomeres, DSBs, Microtubules and Motors

**DOI:** 10.3389/fgene.2018.00157

**Published:** 2018-04-30

**Authors:** Karim Mekhail

**Affiliations:** ^1^Department of Laboratory Medicine and Pathobiology, University of Toronto, MaRS Centre, Toronto, ON, Canada; ^2^Canada Research Chairs Program, University of Toronto, Toronto, ON, Canada

**Keywords:** nuclear organization, DSB mobility, telomeres, DSB repair, kinesin, microtubules, chromatin remodeling, heterochromatin

Reason for corrigendum

The statement that “The MSD y-axis is log scaled” should be added to the end of the figure legend of Figure 1B.

The original article has been updated.

## Conflict of interest statement

The author declares that the research was conducted in the absence of any commercial or financial relationships that could be construed as a potential conflict of interest.

